# Exogenous IL-33 overcomes T cell tolerance in murine acute myeloid leukemia

**DOI:** 10.18632/oncotarget.11179

**Published:** 2016-08-10

**Authors:** Lei Qin, Donye Dominguez, Siqi Chen, Jie Fan, Alan Long, Minghui Zhang, Deyu Fang, Yi Zhang, Timothy M. Kuzel, Bin Zhang

**Affiliations:** ^1^ Biotherapy Center, The First Affiliated Hospital of Zhengzhou University, Zhengzhou, 450052, Henan, China; ^2^ Robert H. Lurie Comprehensive Cancer Center, Department of Medicine-Division of Hematology/Oncology, Northwestern University Feinberg School of Medicine, Chicago, IL 60611, USA; ^3^ Department of Pathology, Northwestern University Feinberg School of Medicine, Chicago, IL 60611, USA

**Keywords:** IL-33, ST2, tolerance, AML, CD8^+^ T cells

## Abstract

Emerging studies suggest that dominant peripheral tolerance is a major mechanism of immune escape in disseminated leukemia. Using an established murine acute myeloid leukemia (AML) model, we here show that systemic administration of recombinant IL-33 dramatically inhibits the leukemia growth and prolongs the survival of leukemia-bearing mice in a CD8^+^ T cell dependent manner. Exogenous IL-33 treatment enhanced anti-leukemia activity by increasing the expansion and IFN-γ production of leukemia-reactive CD8^+^ T cells. Moreover, IL-33 promoted dendritic cell (DC) maturation and activation in favor of its cross presentation ability to evoke a vigorous anti-leukemia immune response. Finally, we found that the combination of PD-1 blockade with IL-33 further prolonged the survival, with half of the mice achieving complete regression. Our data establish a role of exogenous IL-33 in reversing T cell tolerance, and suggest its potential clinical implication into leukemia immunotherapy.

## INTRODUCTION

Leukemia cells express tumor-associated antigen (TAA) that can trigger anti-leukemia immune responses [1, 2]. The most salient evidence is for the potential of the immune system to eradicate acute myeloid leukemia (AML) lies in the graft-versus-leukemia effect associated with allogeneic hematopoietic stem cell transplantation [3]. Although our knowledge of the potential immunotherapy strategies to control AML is still evolving, it is clear that leukemia cells have evolved a number of different immunosuppressive mechanisms [4] to evade these otherwise anti-leukemia responses. These include induction and/or expansion of immunosuppressive cells [regulatory T cells (Tregs) and myeloid derived suppressor cells (MDSC)], up-regulation of T-cell co-inhibitory molecules such as programmed death 1 (PD-1), just to name a few. Compared to the treatment of solid tumors, the contribution of overcoming tumor-induced immune evasion by immunotherapy in hematological malignancies has been less appreciated [5]. Nevertheless, being a systemic disease, leukemia is distinctly different from solid tumors in terms of the cell origin, growth pattern and microenvironment. Recent studies revealed that the dominant peripheral tolerance of leukemia-specific CD8^+^ T cells is a major mechanism of immune escape in disseminated leukemia [6–10]. Therefore, novel strategies are required to overcome the tolerance and restore the anti-leukemia T cell function.

Interleukin-33 (IL-33) is a new member of the IL-1 family cytokines that is mainly present in the nucleus of stromal cells, such as epithelial and endothelia cells [11]. IL-33 is secreted by passive release from necrotic cells to the extracellular space, where they alert the immune system to a local threat like tissue damage and infection [12]. Therefore, IL-33 is classified as “alarmin” cytokine. Upon secretion, IL-33 binds with the receptor complex containing IL-33 specific receptor ST2 (IL-1RL1) and IL-1 receptor accessory protein (IL-1RAcP) to trigger MyD88-dependent pathways. IL-33 can vigorously induce Th2 type immune responses in these cells and thereby promote the development of Th2-related diseases including asthma, atopic dermatitis and anaphylaxis [13–15]. Conversely, IL-33 can also be host-protective in diseases like helminth infection [16] and atherosclerosis [17] by reducing inflammation. Therefore, IL-33 can be either pro-inflammatory or anti-inflammatory, depending on the disease entity. Besides its role in Th2 response, IL-33 has been implicated in promoting Th1 type immune response [18], and enhancing CD8^+^ T cell antiviral responses [19]. Very recently, an IL-33 mediated antitumor effect was observed in murine solid tumor models [20–22]. However, the role of IL-33 in hematological malignancies such as leukemia remains largely unknown.

In this study, we provided the first evidence that systemic administration of recombinant IL-33 dramatically inhibits leukemia growth and prolonged the survival associated with augmented anti-leukemia immune responses of CD8^+^ T cells. Importantly, IL-33 treatment effectively licensed DCs from tumor-bearing mice to overcome the tolerance of leukemia-specific CD8^+^ T cells by inducing expression of different costimulatory molecules. Furthermore, IL-33 treatment significantly improved the efficacy of PD-1 blockade, leading to complete regression of leukemia in half of treated mice, suggesting a viable new combination approach that can be readily translated into the clinic to reverse leukemia-reactive T cell tolerance in promoting leukemia immunotherapy.

## RESULTS

### Exogenous IL-33 treatment alone leads to delayed leukemia development and improved overall survival in the aggressive C1498 AML model

**Figure 1:**
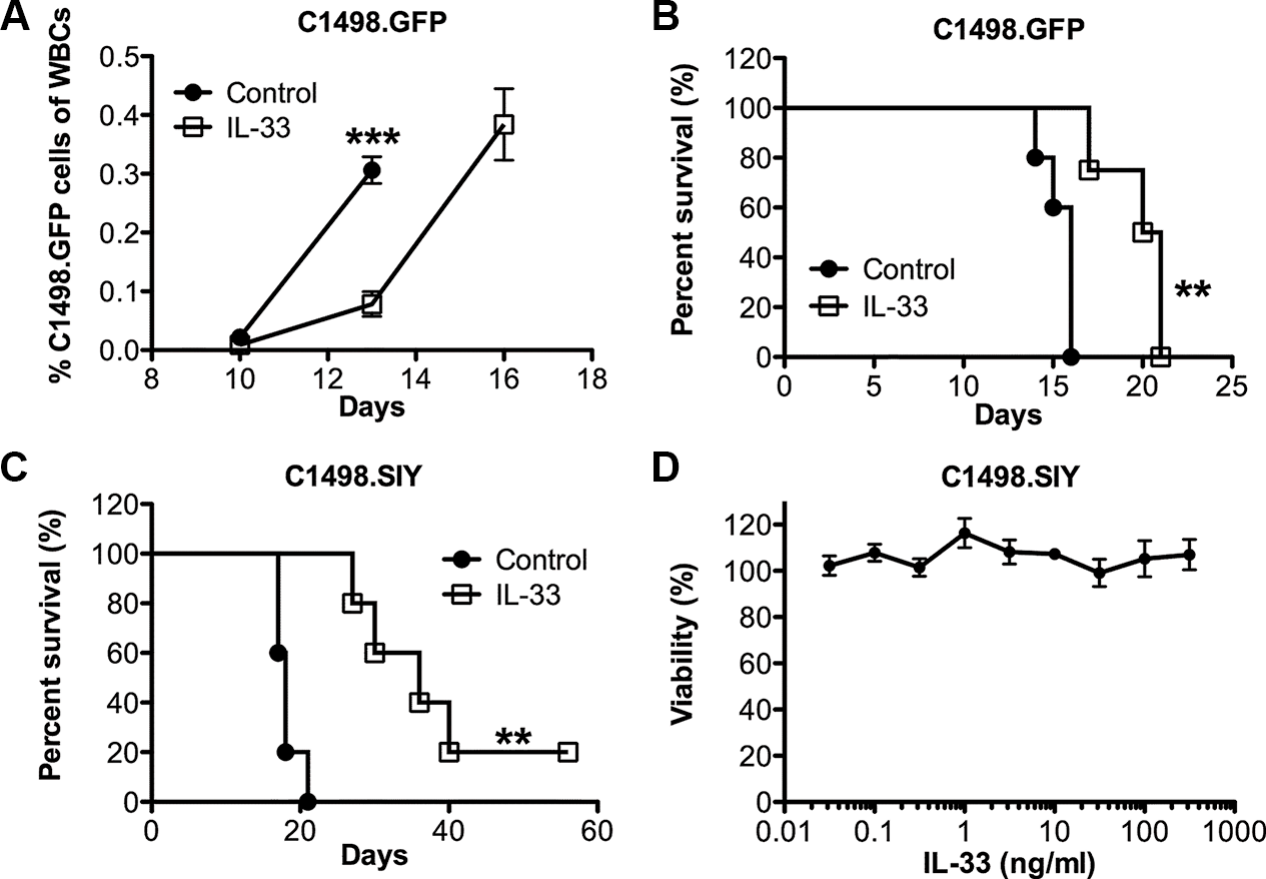
Systemic administration of recombinant IL-33 extends the life span of AML-bearing mice C57BL/6 mice were i.v. injected with C1498.GFP cells and treated with recombinant IL-33 (1 μg/mouse) in PBS or PBS alone i.p. daily starting the following day for 16 days. (**A**) Peripheral blood was drawn on day 10, 13, 16, and the percentage of leukemia cells among total white blood cells was determined by GFP expression using flow cytometry (*n* = 5). (**B**) Mice survival was monitored in the C1498.GFP model (*n* = 5). (**C**) Mice were challenged i.v. with GFP expressing C1498.SIY cells and received IL-33 or control PBS treatment for 26 days, and survival was assessed (*n* = 5). (**D**) IL-33 does not inhibit leukemia cell proliferation *in vitro*. C1498.SIY cells were incubated with different concentration of recombinant IL-33 for 48 hours, and cell viability was quantified. Each point represents the mean ± SEM of 3 replicates. ***p < 0.001. **p < 0.01. Data are representative of 2 independent experiments.

To examine the potential anti-leukemia effect of exogenous IL-33, we challenged the C57BL/6 mice with the murine acute myeloid leukemia cell line C1498.GFP. The GFP expression allows us to monitor the leukemia development in peripheral blood and its target organs. Recombinant IL-33 (1 μg/mouse) or PBS as control was given intraperitoneally (i.p.) daily after intravenous C1498. GFP cell challenge. The leukemia developed aggressively in the control group as reflected by the rapid expansion of GFP^+^ cells among total white blood cells (Figure 1A). IL-33 treatment dramatically inhibited C1498.GFP growth. On day 13, the leukemia burden in control mice was three times more than that in IL-33-treated mice. By day 16, all mice from control group were dead of leukemia. In contrast, IL-33 treatment significantly extended the life span of leukemia-bearing mice from a median survival 16 days to 21 days (Figure 1B). We subsequently tested the effect of IL-33 using GFP expressing immunogenic C1498.SIY cells in the same setting. Similarly, IL-33 treatment prolonged the life span of leukemia-bearing mice from a median survival 18 days to 36 days, while being more effective in inhibiting the growth of C1498. SIY cells than parental C1498.GFP cells (Figure 1C). Thus, the data indicate that the efficacy of exogenous IL-33 administration appears to depend in part on tumor immunogenicity, which was also supported by the fact that IL-33 treatment did not directly affect leukemia cell viability *in vitro* (Figure 1D).

### Exogenous IL-33 boosts anti-leukemia activity in a CD8^+^ T cell dependent manner

**Figure 2:**
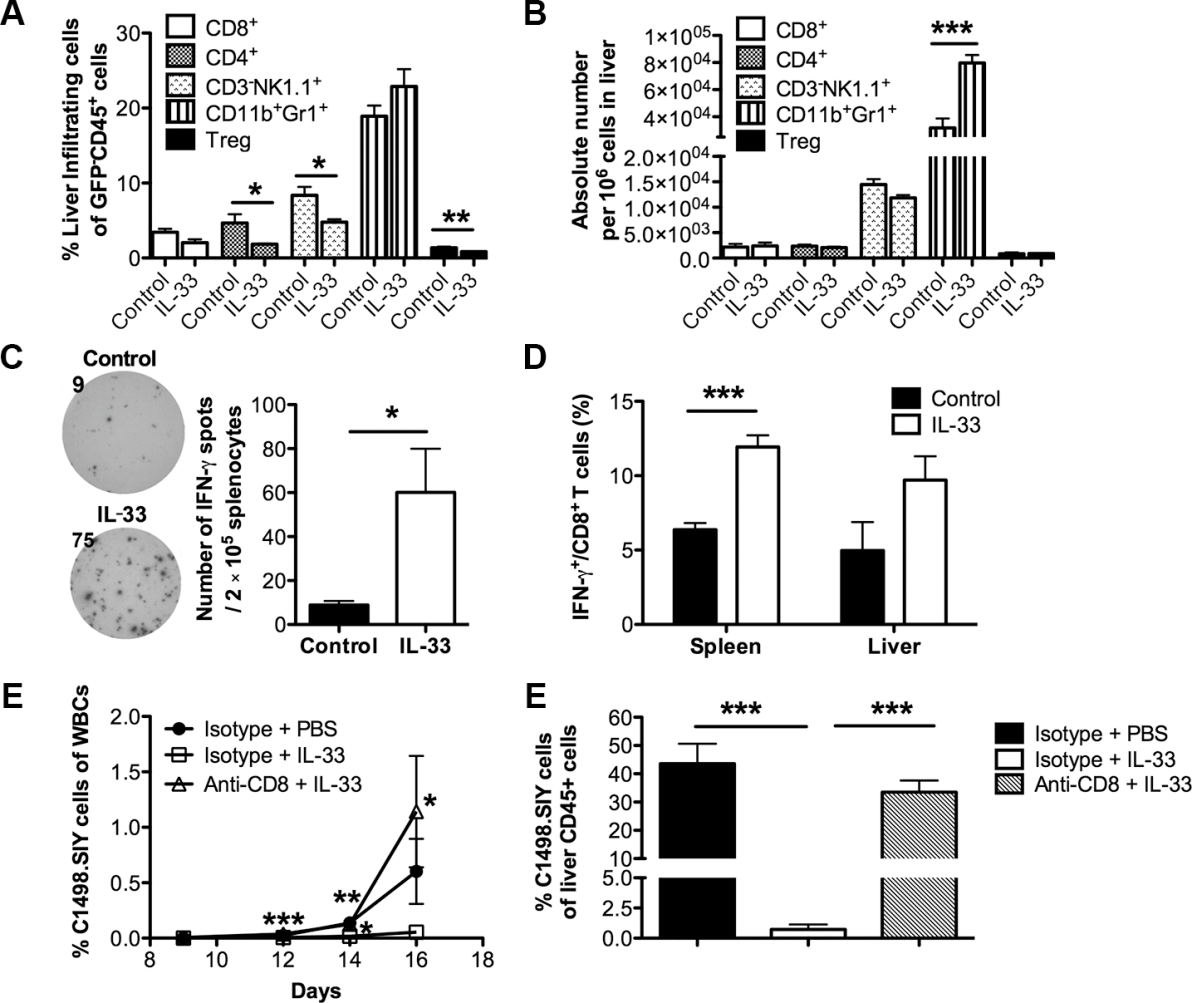
Exogenous IL-33 elicits anti-leukemia activity in a CD8^+^ T cell dependent manner Mice were challenged i.v. with GFP expressing C1498.SIY cells and treated with recombinant IL-33 or PBS daily for 16 days. Spleen and liver were then harvested for flow analysis (*n* = 5). (**A**) Percentages of liver-infiltrating CD8^+^, CD4^+^, CD3^−^NK1.1^+^, CD11b^+^Gr1^+^, CD4^+^Foxp3^+^ cells among leukocytes (GFP^−^CD45^+^) were determined by flow cytometry. (**B**) Absolute numbers of indicated liver infiltrates were calculated. (**C**) Splenocytes from each leukemia-bearing mouse were stimulated with SIY peptides for 48 hours (*n* = 5). Number of IFN-γ producing cells was determined by IFN-γ ELISPOT assay. Data (mean ± SEM) are representative of 3 independent experiments. (**D**) The frequencies of spleen and liver IFN-γ^+^ cells among CD8^+^ T cells were examined by flow cytometry. To determine the importance of CD8^+^ T cells, CD8^+^ T cells were depleted by i.p. injection of CD8-depleting antibodies every 3 days starting from 1 day prior to C1498.SIY challenge. (**E**) Peripheral blood was drawn from each group on day 9, 12, 14, 16, and the percentage of leukemia cells among total white blood cells was determined by GFP expression using flow cytometry (*n* = 3). ***p < 0.001 (day 12), **p < 0.01 (day 14) and *p < 0.05 (day 16) for **Δ** vs □ *p < 0.05 (day 14) for □ vs ●. (**F**) On day 16, tumor burden in liver was calculated by the percentage of GFP^+^ cells among liver CD45^+^ cells by flow cytometry (*n* = 5). *p < 0.05. **p < 0.01. ***p < 0.001.

Given the strong immunoregulatory roles of IL-33 [23], we initially characterized the immune cell infiltration in liver, which is one of the main target organs of AML cells [24]. On day 16, we found no significant alteration in percentages of CD8^+^ T cells and Gr1^+^CD11b^+^ MDSCs in C1498.SIY-bearing mice (Figure 2A). Although IL-33 treatment lowered the percentages of CD4^+^ T cells, NK cells and CD4^+^Foxp3^+^ Tregs among liver non-leukemia leukocytes (GFP^−^CD45^+^), there were no significant differences in absolute numbers of different immune subsets tested except for an increase of MDSCs (Figure 2B). To investigate whether IL-33 treatment promotes an anti-leukemia immune response, an IFN-γ ELISPOT was conducted using splenocytes from each group 16 days after C1498.SIY challenge. Cells were stimulated with SIY peptides or left unstimulated as negative controls. IL-33 treatment resulted in more IFN-γ spot forming cells whereas only minimal functional responses were detected in the control group (Figure 2C). As expected, IFN-γ spots were barely detected in both groups without SIY stimulation (Supplementary Figure S1). We also found that IL-33 treatment increased the percentage of IFN-γ producing CD8^+^ T cells in both spleen and liver from leukemia-bearing mice by flow cytometry (Figure 2D). The data indicate that IL-33 may improve the overall survival by boosting the anti-leukemia immunity through CD8^+^ T cells. To confirm this further, we depleted CD8^+^ T cells by injecting anti-CD8 antibody starting one day prior to C1498.SIY challenge. CD8^+^ T cell depletion diminished the anti-leukemia effect of exogenous IL-33, as reflected by progressive leukemia burden in peripheral blood (Figure 2E) and liver (Figure 2F) comparable with those of the control PBS group.

### Exogenous IL-33 reverses antigen-specific anti-leukemia CD8^+^ T cell dysfunction

**Figure 3:**
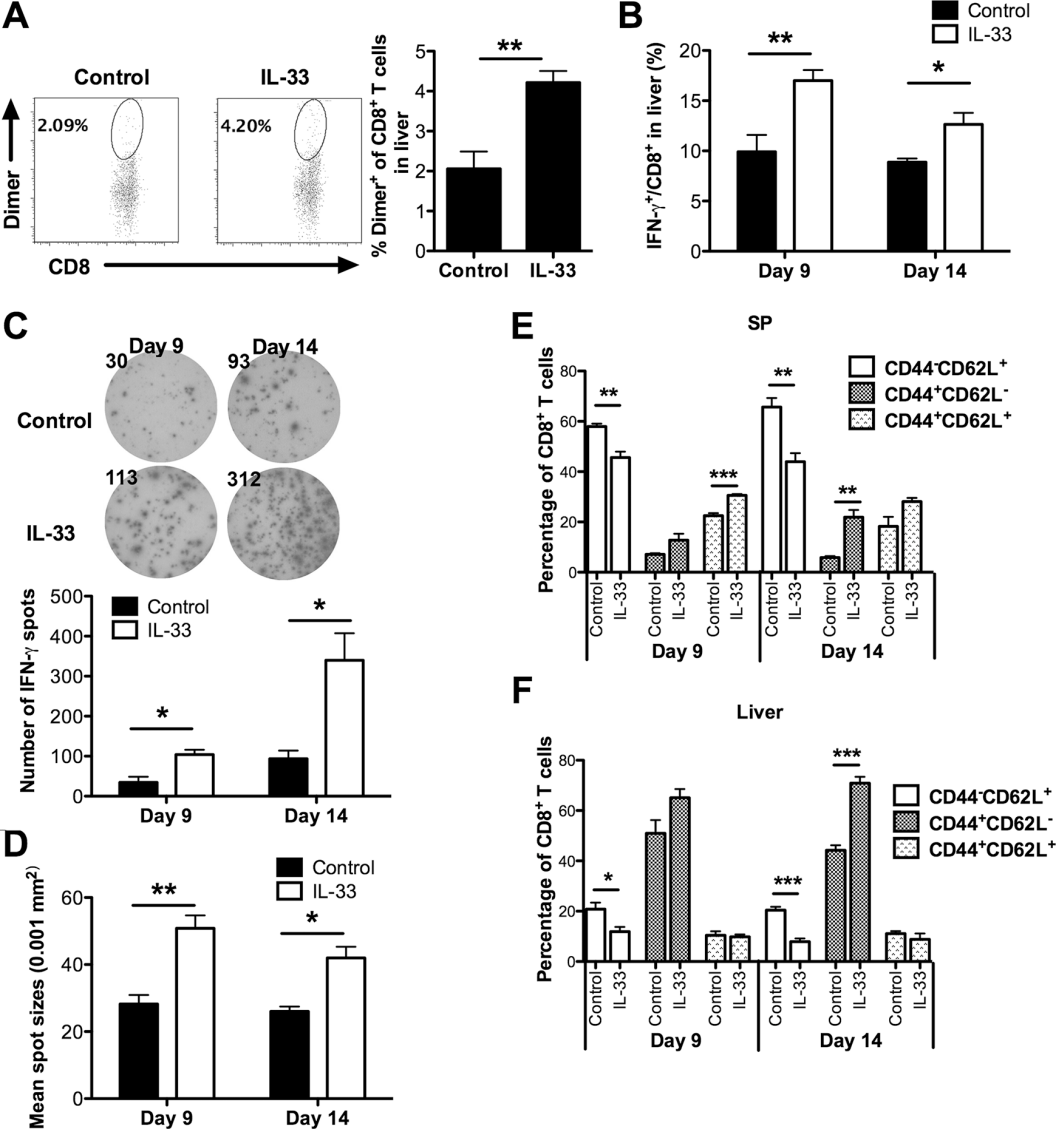
Exogenous IL-33 enhances antigen-specific anti-leukemia CD8^+^ T cell immunity Mice were challenged i.v. with GFP expressing C1498.SIY cells and treated with IL-33 or PBS daily. Spleen and liver were harvested for flow analysis on day 9 and 14. (**A**) The frequency of SIY-specific CD8^+^ T cells in livers was analyzed after staining with H-2K^b^-SIYRYYGL-IgG dimer. The *upper* panel shows representative flow dot plots (day 9), and *lower* panel shows a summary of SIY-specific CD8^+^ T cell percentage (*n* = 5). (**B**) The frequency of IFN-γ^+^ CD8^+^ T cells in liver is summarized. (**C**–**D**) Splenic CD8^+^ T cell function was evaluated by IFN-γ ELISPOT assay. CD8^+^ T cells purified from each group were co-cultured with purified splenic DCs from naïve WT mice at 1:1 ratio in the presence of SIY peptide for 48 hours (*n* = 3). (C) The *upper* panel showed the representative ELISPOT wells, and the *lower* panel showed the average numbers of IFN-γ producing cells. The average size of IFN-γ spots is indicated in (D). The CD44 and CD62L expression on splenic CD8^+^ T cells from spleen (**E**) and liver (**F**) of IL-33- or control PBS-treated leukemia-bearing mice was analyzed by flow cytometry (*n* = 5). *p < 0.05. **p < 0.01. ***p < 0.01.

To further dissect the anti-leukemia effect of IL-33, we examined the frequency and function of effector CD8^+^ T cells at early (day 9) and late (day 14) stages. Dimer staining showed a significantly higher number of SIY-reactive liver CD8^+^ T cells in IL-33-treated mice than in control mice at early stages (Figure 3A). Moreover, IL-33 treatment increased the percentage of IFN-γ producing CD8^+^ T cells in liver of these leukemia-bearing mice at both early and late stages (Figure 3B).

To examine the effect of exogenous IL-33 solely on leukemia-reactive CD8^+^ T cells, splenic CD8^+^ T cells purified from either PBS- or IL-33-treated C1498.SIY bearing mice were stimulated with naïve WT DCs pulsed with SIY peptides for detection of IFN-γ production by ELISPOT. As shown in Figure 3C, CD8^+^ T cells from the control PBS-treated group failed to generate a functional SIY-specific T cell response, confirming the notion that induction of peripheral T cell tolerance is a common mechanism of immune evasion in hosts with disseminated AML [5, 10]. In contrast, a greater number of IFN-γ producing cells were observed in IL-33-treated mice on both day 9 and day 14. The size of IFN-γ spots of CD8^+^ T cells from IL-33-treated mice was also larger than that from PBS-treated control mice (Figure 3D).

To characterize the differentiation status of CD8^+^ T cells, we examined the expression of CD44 and CD62L in spleen and liver. We found that IL-33 drove significantly more splenic CD8^+^ T cells to an activated phenotype on both day 9 and day 14, as indicated by decreased percentages of naive T cells (CD44^−^CD62L^+^) in IL-33-treated mice (Figure 3E). Meanwhile, IL-33 treatment increased the proportion of central memory CD8^+^ T cells (CD44^+^CD62L^+^) on day 9, and more CD8^+^ T cells displayed an effector memory phenotype (CD44^+^CD62L^−^) on day 14 in spleen (Figure 3E). Similarly, we found that fewer liver CD8^+^ T cells displayed a naïve phenotype on day 9 and day 14 following IL-33 treatment (Figure 3F). IL-33 treatment also significantly increased the percentage of effector memory liver CD8^+^ T cells on day 14 (Figure 3F), indicating a role of exogenous IL-33 in promoting rapid expansion of the effector memory pool, consistent with the results from solid tumor models [21, 22].

### Exogenous IL-33 helps overcome peripheral T cell tolerance through promoting antigen-specific CD8^+^ T cell proliferation and function

**Figure 4:**
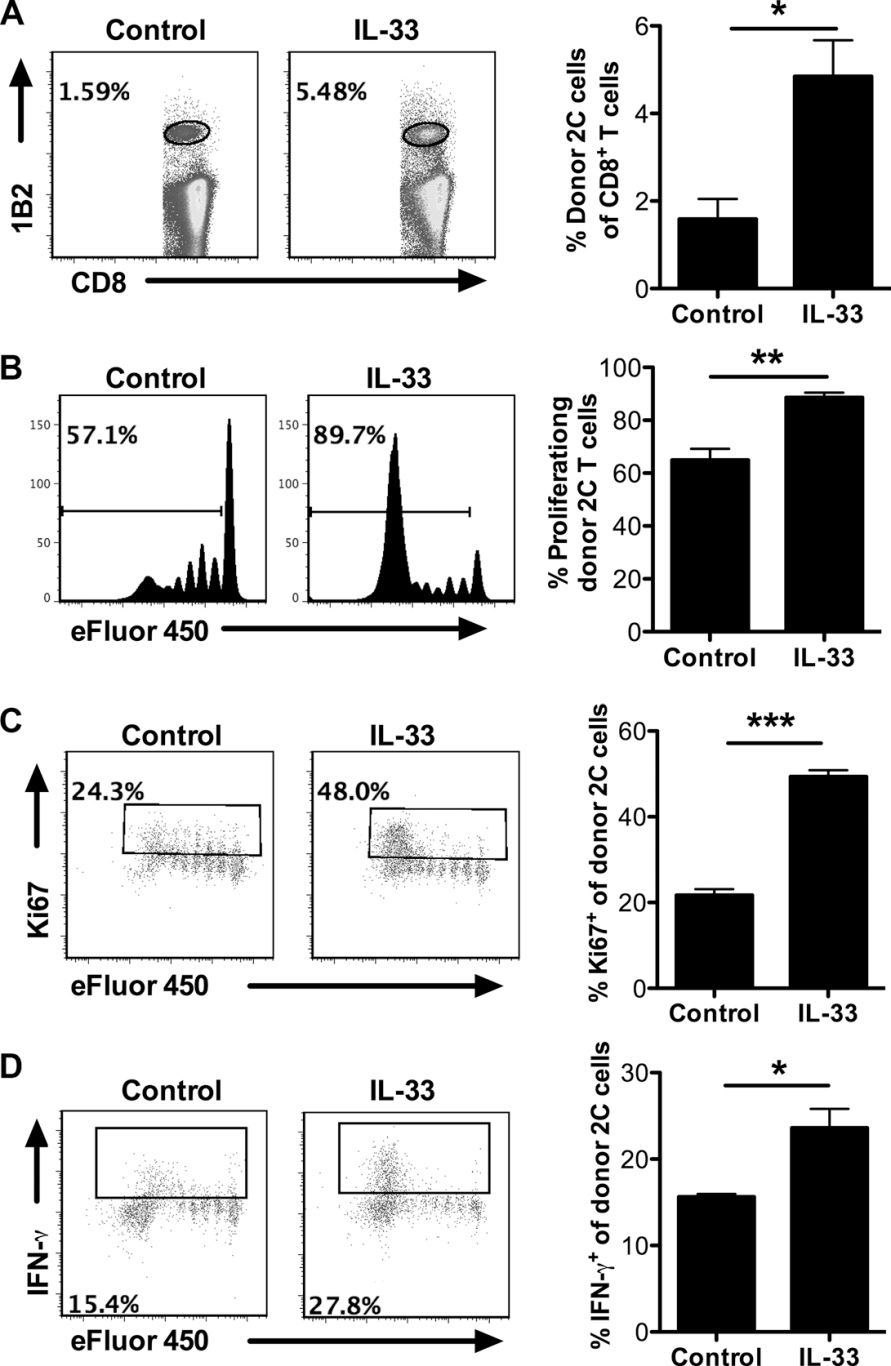
Exogenous IL-33 promotes antigen-specific CD8^+^ T cell proliferation and function after adoptive transfer Efluor450-labeled SIY-specific 2C CD8^+^ T cells were adoptively transferred to mice 5 days after C1498.SIY i.v. injection. On the same day with T cell transfer, mice (*n* = 3) were treated daily with IL-33 (1 μg/mouse) or PBS as control for 7 days. Spleens were harvested for analysis. (**A**) The frequency of donor 2C T cells (1B2^+^CD8^+^) among the entire CD8^+^ T cell population. (**B**) Efluor450 dilution of transferred 2C T cells. (**C**) The frequency of Ki67^+^ cells among transferred 2C T cells. (**D**) The frequency of IFN-γ^+^ cells among transferred 2C T cells. *p < 0.05. **p < 0.01. ***p < 0.01.

To further determine the ability of exogenous IL-33 to overcome the peripheral T cell tolerance in leukemia-bearing hosts, we carried out an adoptive transfer model using SIY-specific 2C TCR transgenic CD8^+^ T cells. Efluor450-labeled 2C T cells were i.v. injected into mice 5 days after C1498.SIY i.v. injection. IL-33 treatment significantly increased the proportion of 2C T cells in total CD8^+^ T cells (Figure 4A). More importantly, 2C T cells from IL-33-treated mice proliferated vigorously and the majority underwent division (eFluor450 negative), whereas 2C T cells from PBS-treated mice proliferated to a lesser extent and the majority remained undivided (eFluor450 positive) (Figure 4B). Similar results were also observed in this setting using cell proliferation marker Ki67 analysis (Figure 4C), further confirming the ability of IL-33 to rescue antigen-specific CD8^+^ T cell proliferation. In addition, IL-33 appeared to affect minimally the apoptotic status of those 2C T cells (Supplementary Figure S2). Functional analysis of 2C T cells revealed increased percentage of IFN-γ producing cells in response to IL-33 treatment (Figure 4D).

### Exogenous IL-33 licenses DC activation and enhances its cross-priming to leukemia-reactive CD8^+^ T cells

**Figure 5:**
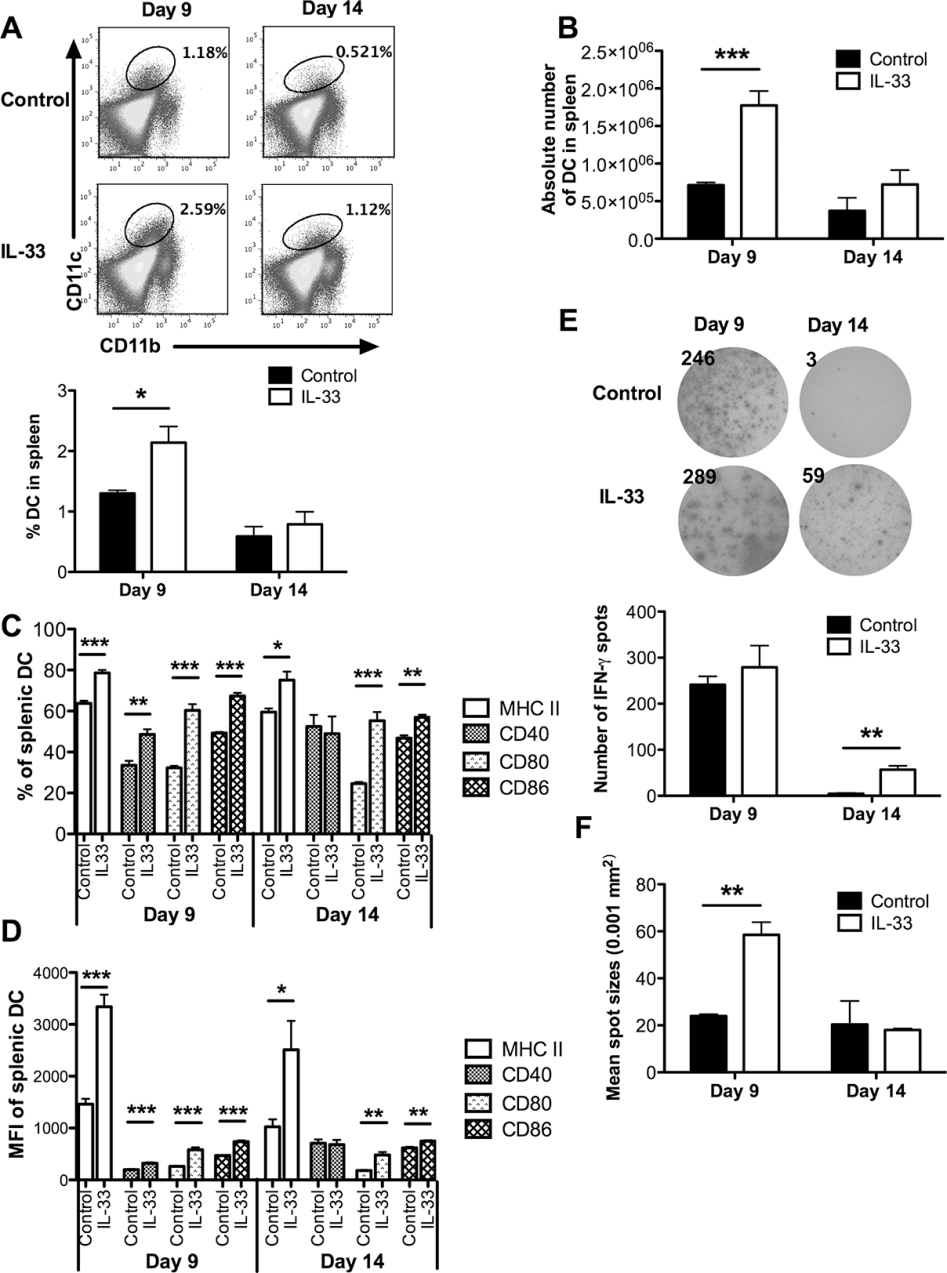
Exogenous IL-33 reverses DC tolerance by inducing costimulatory molecule expression, and mediates DC licensing to prime leukemia-reactive CD8^+^ T cells Mice challenged with C1498.SIY cells were treated daily with IL-33 or PBS. Splenic DCs were analyzed on day 9 and 14. (**A**) Representative flow dot plots showed the frequency of splenic CD11b^+^CD11c^+^ DCs (*upper*) that was summarized in the bar graph (*lower, n* = 5). (**B**) The absolute number of splenic CD11b^+^CD11c^+^ DCs (*n* = 5). The percentage (**C**) and MFI (**D**) of MHC II, CD40, CD80 and CD86 expression were determined in splenic DCs (*n* = 5). CD11c^+^ DCs purified from each group were co-cultured with CD8^+^ T cells from naïve 2C mice at 1:1 ratio for 48 hours. DC cross-priming ability was evaluated by IFN-γ ELISPOT assay (*n* = 3). (**E**) The *left* panel showed the representative ELISPOT wells, and the *right* panel showed the average numbers of IFN-γ producing cells. The average size of IFN-γ spots was indicated in (**F**). *p < 0.05. **p < 0.01. ***p < 0.001.

As host DCs may mediate T cell tolerance in AML [21, 22], we hypothesized that exogenous IL-33 may work through DCs to reverse leukemia-reactive CD8^+^ T cell dysfunction. In support of this, we found a significantly higher proportion as well as absolute number of DCs in IL-33-treated leukemia-bearing mice compared with control mice on day 9, but not on day 14 (Figure 5A and 5B). The MHC class II and costimulatory molecules (CD40, CD80, CD86) expressed on DCs play a key role in triggering efficient antigen-specific T cell responses. Using flow cytometry, we found that IL-33 treatment significantly up-regulated the percentages of MHC II^+^, CD80^+^ and CD86^+^ splenic DCs on both day 9 and day 14 (Figure 5C and Supplementary Figure S3). In addition, the percentage of CD40^+^ splenic DCs was also increased in response to IL-33 treatment on day 9 (Figure 5C). Similar trends were observed for MFI of these markers (Figure 5D).

To test whether IL-33 treatment licenses DCs to efficiently prime leukemia-reactive CD8^+^ T cells, splenic CD11c^+^ cells purified from either PBS- or IL-33-treated C1498.SIY-bearing mice were cocultured with SIY-specific 2C CD8^+^ T cells for detection of IFN-γ production by ELISPOT. Despite finding no difference in the number of IFN-γ producing cells between IL-33-treated and control mice, the size of IFN-γ spots from IL-33-treated mice was significantly larger than that from control mice on day 9 (Figure 5E). On day 14, IFN-γ production by 2C CD8^+^ T cells was barely detected from control mice (Figure 5E), suggesting a tolerogenic feature of DCs at the later stages of leukemia progression. However, DCs from IL-33-treated mice retained an ability to trigger IFN-γ production by these 2C CD8^+^ T cells (Figure 5E).

Given the importance of CD8^+^ DCs in the immune recognition of AML [25], we examined the CD8^+^ DC accumulation in leukemia-bearing mice following IL-33 treatment. IL-33 treatment reduced the percentage of splenic CD8^+^ DCs (Supplementary Figure S4A), but there was no significant difference in the absolute number of splenic CD8^+^ DCs between groups (Supplementary Figure S4B). Further, IL-33 treatment preferentially increased CD80 expression on these CD8^+^ DCs (Supplementary Figure S4C).

### IL-33 treatment upregulates PD-1/PD-L1 expression *in vivo*, and combination of PD-1 blockade and IL-33 treatment further improves survival of leukemia-bearing mice

**Figure 6:**
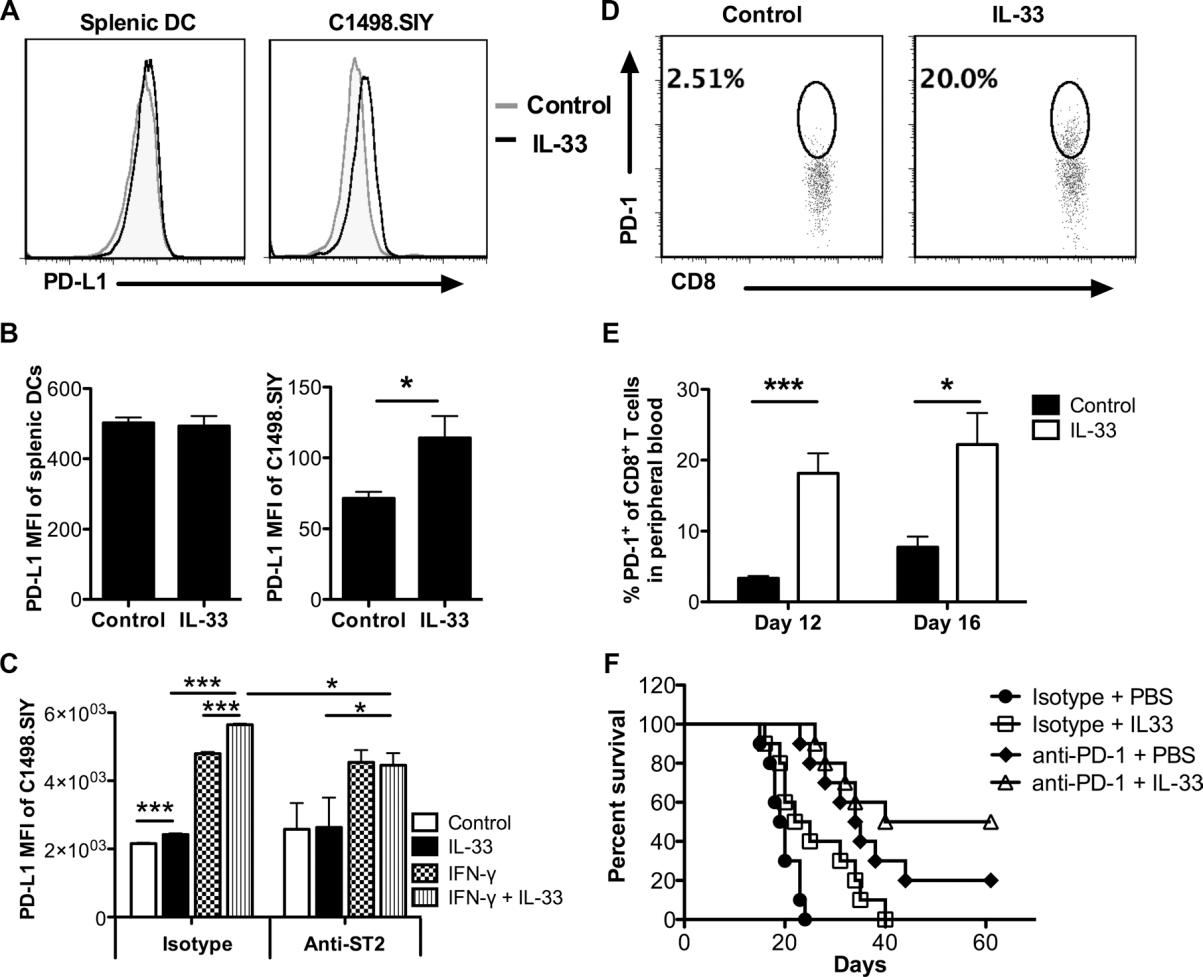
IL-33 treatment induces PD-1/PD-L1 expression *in vivo*, and combination of PD-1 blockade and IL-33 treatment further improves survival of AML-bearing mice Mice were challenged i.v. with C1498.SIY cells and treated with IL-33 or PBS daily. On day 16, the PD-L1 expression on splenic DCs and C1498.SIY cells from liver was evaluated by flow cytometry (*n* = 3). The representative histograms were shown in (**A**), and median fluorescence intensity (MFI) of PD-L1 was summarized in (**B**). (**C**) C1498.SIY cells were treated *in vitro* with IFN-γ (20 ng/ml), IL-33 (1 ng/ml), both or left untreated in the absence or presence of anti-ST2 (10 ug/ml) blocking antibodies for 24 hours. The MFI of PD-L1 expression on C1498.SIY cells was analyzed by flow cytometry (*n* = 3). Data are representative of 2 independent experiments. Peripheral blood was drawn from PBS or IL-33 treated C1498.SIY-bearing mice (*n* = 5) on day 12 and 16. The percentage of PD-1^+^ cells among peripheral CD8^+^ T cells was determined by flow cytometry in (**D**) and summarized in (**E**). (**F**) WT mice were challenged i.v. with C1498.SIY cells. The anti-PD-1 (150 μg/mouse) or isotype control antibodies were administered i.p. every 3 days for 5 times. IL-33 (1 μg/mouse) were injected daily for 18 days. IL-33 alone or anti-PD-1 alone prolonged the life span of C1498.SIY bearing mice (*p < 0.05 for □ vs ●, *** *p* < 0.001 for □ vs ●). The combination treatment of anti-PD-1 and IL-33 significantly improved mice survival compared with IL-33 treatment alone (**p < 0.01 for Δ vs □) (*n* = 10 per group).

The blockade of PD-1/PD-L1 interaction has demonstrated highly durable response rates in certain types of solid tumors and hematological malignancies [26, 27]. To check the effect of IL-33 on PD-1/PD-L1 pathway in C1498.SIY leukemia model, we initially examine the PD-L1 expression on leukemia cells and DCs following IL-33 treatment. Sixteen days after C1498.SIY challenge, significantly higher PD-L1 expression levels were observed on liver C1498.SIY cells in IL-33-treated group compared with those in control group (Figure 6A, 6B). However, no difference of PD-L1 expression was detected on splenic DCs between groups (Figure 6A, 6B). As IFN-γ was reported to induce PD-L1 expression [28, 29], we hypothesized that IL-33 may work together with IFN-γ to upregulate PD-L1 expression on C1498.SIY cells. To test this hypothesis, C1498.SIY cells were treated with IL-33, IFN-γ, or both *in vitro*. Similar to IFN-γ, IL-33 alone upregulated PD-L1 expression but to a lesser extent (Figure 6C). Combination treatment with IFN-γ plus IL-33 induced the highest expression level of PD-L1, greater than either treatment alone (Figure 6C). When blocking IL-33 receptor using ST2 neutralizing antibodies, the difference between IL-33-treated and untreated controls, as well as between IFN-γ alone and combination was abrogated. However, the difference between IFN-γ alone and untreated controls remained unchanged (Figure 6C), suggesting a direct requirement of ST2 for PD-L1 induction by IL-33 but not IFN-γ. In addition, PD-1 expression on CD8^+^ T cells in peripheral blood was also induced by exogenous IL-33 (Figure 6D, 6E).

Given the induction of IL-33 on PD-1/PD-L1 expression, we examined the anti-leukemia activity of combination treatment with PD-1 blockade plus IL-33 administration. As expected, combination treatment prolonged the life span of C1498.SIY bearing mice compared with each therapy alone (Figure 6F). Of note, half of treated mice from combination therapy showed long-term survival, suggesting a novel combination immunotherapy strategy against aggressive AML.

## DISCUSSION

The role of IL-33 in hematological malignancies such as AML has not been established. We show in this study that systemic administration of recombinant IL-33 inhibited leukemia growth and prolonged the survival of C1498 leukemia-bearing mice in a CD8^+^ T cell dependent manner. This was associated with enhanced anti-leukemia CD8^+^ T cell activity, and DC maturation and activation. Furthermore, in this aggressive AML model, long-term survival was observed in half of mice with combined exogenous IL-33 treatment and PD-1 blockade, pointing to a clinically translatable regimen for treating advanced AML patients.

Recent studies from transplantable solid tumor models have indicated a direct role of exogenous IL-33 in promoting antitumor CD8^+^ T cell immunity using either IL-33 transgenic mice [20], IL-33 DNA as vaccine adjuvant [22], or IL-33 expressing tumor cells [21]. Consistent with these studies, our data extend these findings to an AML model but using recombinant IL-33. Our data suggest that reversing DC dysfunction by exogenous IL-33 treatment also contributes to the enhanced leukemia-reactive T cell-mediated antitumor immune response. On the other hand, it is worth mentioning that the IL-33/ST2 signaling has been reported to contribute to CML progenitor growth [30] and the pathogenesis of myeloproliferative neoplasms [31], but lacked assessment of host immune responses. Interestingly, the amount of exogenous IL-33 we used is about 5-50 times higher than that from these reports. Furthermore, other studies showed that the IL-33/ST2 axis increased effector T-cell responses to worsen acute GVHD [32], and ST2 blockade reduced GVHD severity and mortality while preserving graft-versus-leukemia activity [33]. Therefore, the biological activities of IL-33 appear to be context-dependent, and could be determined by the amount and primary target cells of IL-33 in different models.

Unlike solid tumors that are mainly constrained locally, AML is a disseminated (systemic) disease. Seminal results from Kline lab recently demonstrate that systemic challenging with AML cells (i.v.) elicits a dominant peripheral T cell tolerance particularly in the C1498 model compared with local challenge (s.c.) [6]. Moreover, this T cell dysfunction requires DCs but is independent of Tregs or MDSC [6]. The ability of DCs to activate T cells is determined by their maturation status. In the absence of inflammatory stimuli (i.e., under steady-state conditions), DCs are quiescent and often maintain peripheral tolerance to self-antigens. Due to the disseminated nature of AML, and the lack of a classical tumor-draining lymph nodes, DCs could be exposed to insufficient “danger signals” from AML cells and thereby be incapable of effectively cross-priming leukemia-reactive T cells [5, 10]. The net effect is the induction of peripheral T cell tolerance to AML. In our study, we indeed confirmed that DCs became tolerogenic when AML progressed to a late stage (day 14) as reflected by the loss of ability to cross-prime leukemia-reactive T cells. However, exogenous IL-33 treatment enhanced DC maturation by the up-regulation of several costimulatory molecules, consistent with other studies in the non-tumor-bearing settings [34, 35]. As a result, these DCs were able to break the tolerance and induce effective anti-leukemia CD8^+^ T cell response. It is thus conceivable that exogenous IL-33 functions as an “alamin”, a danger signal to promote DC activation and licensing.

We demonstrate that IL-33-mediated anti-leukemia activity depends upon CD8^+^ T cells. Phenotypically, IL-33 treatment decreased the proportion of naïve (CD44^−^CD62L^+^) CD8^+^ T cells, and increased the proportion of central memory (CD44^+^CD62L^+^) or effector memory (CD44^+^CD62L^−^) CD8^+^ T cells. These results are in line with the observations that IL-33 can enhance effector memory T cell expansion in solid tumor models [19], further supporting the concept that IL-33-induced effector memory CD8^+^ T cells may be important for effective tumor clearance. However, we and others [21] found that IL-33 also increased host MDSC population. A recent study reported that IL-33 in tumor microenvironment reduced the apoptosis and maintained the survival of MDSCs through induction of autocrine secretion of GM-CSF [36]. Thus, it is conceivable that the efficacy of IL-33 therapy may be further improved by multiple methods of inhibiting MDSCs that are currently under clinical development.

The PD-1/PD-L1 pathway plays a key role in immune evasion in AML [24, 37, 38]. Interestingly, we found that IL-33 treatment promoted anti-leukemia activity but induced the PD-L1 expression on leukemia cells and PD-1 expression on CD8^+^ T cells. As PD-1 can be up-regulated after TCR-mediated activation or common-chain cytokine stimulation [39], it is possible that PD-1 upregulation on T cells may work as a negative feedback mechanism for IL-33-induced T cell activation. On the other hand, IL-33 can directly stimulate T cell production of IFN-γ that may in turn induce PD-L1 expression on leukemia cells. Understanding the mechanisms by which IL-33 influences the PD-1/PD-L1 pathway warrants further investigation. Importantly, we showed that combining IL-33 treatment with PD-1/PD-L1 blockade conferred superior anti-leukemia activity compared to either therapy alone, leading to even long-term tumor-free survival in half of these leukemia-bearing mice. Targeting PD-1/PD-L1 signaling in clinical trials has shown clinical activity not only in solid tumors but also in hematological malignancies. Therefore, analyzing the role of IL-33 in potentiating the therapeutic targeting of PD-1/PD-L1 signaling in AML could be of great value in the clinic.

## MATERIALS AND METHODS

### Mice and tumor cell lines

C57BL/6 mice, 6–8 weeks old, were purchase from either Jackson Laboratory or Harlan Laboratory. The 2C TCR transgenic mice were generously provided by Dr. Hans Schreiber (University of Chicago). Mice were used according to the protocols approved by institutional animal use committee at Northwestern University. The murine acute myeloid leukemia cell line C1498.GFP and C1498.SIY were cultured in DMEM supplemented 10% fetal bovine serum at 37°C in a 5% CO_2_ incubator. C1498.GFP cells and C1498.SIY cells were generated by retroviral transduction of parental C1498 cells with pLEGFP plasmid, or pLEGFP plasmid expressing cDNA for the SIYRYYGL (SIY) model peptide antigen in frame to eGFP [6]. Both leukemia cells lines were generously provided by Dr. Justin Kline (University of Chicago).

### Tumor challenge and treatment

C1498.GFP and C1498.SIY cells were washed and resuspended in sterile PBS, and 2 × 10^6^ cells were intravenously injected (i.v.) into the lateral tail vein of mice. For IL-33 treatment, mice received daily i.p. injection of recombinant murine IL-33 (BioLegend) dissolved in PBS or PBS alone as control starting from one day after tumor challenge. Mice were given recombinant murine IL-33 (1 μg/mouse) dissolved in PBS or PBS alone as control daily through i.p. injection, starting from one day following the tumor challenge. CD8^+^ T cell depletion was achieved as described previously [40]. Briefly, anti-CD8 (clone 53–6.7, 200 μg/mouse; BioXCell) or isotype control antibodies were injected i.p. every 3 days starting from one day prior to tumor challenge. For combination therapy, anti-PD-1 blocking antibodies (clone RMP1-14, 150 μg/mouse; BioXCell) or isotype control antibodies were administrated i.p. every 3 days for 5 times starting on the same day of the 1^st^ IL-33 treatment.

### Adoptive transfer of 2C T cells into C1498.SIY bearing mice

C1498.SIY cells (2 × 10^6^) were i.v. injected into C57BL/6 mice. On day 5, SIY antigen-specific CD8^+^ T cells from 2C TCR transgenic mice (2C T cells) were purified using CD8^+^ T cell enrichment kit (StemCell Technologies) and labeled with eFluor450 (eBioscience) as described by the manufacture. 4 × 10^6^ eFluor450-labeled 2C T cells were i.v injected into the C1498.SIY bearing mice. Starting from day 5, mice were treated with recombinant murine IL-33 (1 μg/mouse) dissolved in PBS or PBS daily for 7 days. A cohort of mice that received all the above procedure except C1498.SIY challenge were used as negative controls. On day 12, spleens were harvested for analysis of transferred 2C T cells. 2C T cells were identified by staining with a 2C TCR-specific 1B2 antibody together with anti-CD8 antibody. The absolute number, percentage, eFluor450 dilution, Ki67 expression, IFN-γ expression, apoptotic percentage of 2C T cells were analyzed by flow cytometry.

### Flow cytometry

The flow antibodies were purchased from Biolegend and eBioscience. PE soluble dimeric mouse H-2Kb: Ig fusion protein (DimerX) was from BD Biosciences. Dimer staining, Annexin V staining, Foxp3 and Ki67 staining, and intracellular IFN-γ staining were performed as published previously [40]. The samples were measured by MACSQuant Analyzer (Miltenyi Biotec) and were analyzed using FlowJo software. When analyzing liver leukocytes, the gate was set on CD45^+^GFP^−^ cells to exclude the non-leukocyte cells and tumor cells.

### IFN-γ ELISPOT assay

ELISPOT was performed using eBioscience mouse IFN-γ ELISPOT Ready-SET-Go kit according to the manufacturer's protocol. To evaluate the IFN-γ production using splenocytes, 2 × 10^5^ splenocytes from each individual mouse were seeded into the wells with SIY peptide (5 μM) or without SIY peptide as negative control at 37°C for 48 hours. For evaluation of *ex vivo* CD8^+^ T cell IFN-γ production, purified CD8^+^ T cells (10^5^) from individual C1498.SIY bearing mouse were co-cultured with splenic DCs from naïve WT mice at 1:1 ratio in the presence of SIY (5 μM) peptide for 48 hours. The CD8^+^ T cells alone were used as controls. For evaluation of *ex vivo* DC cross-priming ability, splenic DCs (10^5^) purified from each group were co-cultured with CD8^+^ T cells from naïve 2C mice at 1:1 ratio for 48 hours. The naïve 2C CD8^+^ T cells alone were used as controls. The number and diameter of IFN-γ spots were analyzed using ImmunoSpot software.

### Cell viability assay

Viability of C1498.SIY cells was examined using Cell Titer 96 Aqueous One Solution cell proliferation assay kit (Promega). The percentage of cell viability was calculated using the following formula: Viability (%) = (OD_IL-33 treated_ – OD_background_)/(OD_untreated_ – OD_background_) × 100.

### Statistics

A two-tailed Student's *t* test was used to compare difference between two groups. For survival difference between groups, the log-rank test was use. A *p value* < 0.05 was considered statistically significant. Data are presented as mean ± SEM in all experiment.

## SUPPLEMENTARY MATERIALS FIGURES


