# Supplementary material for: Exogenous IL-33 overcomes T cell tolerance in murine acute myeloid leukemia
Source: Oncotarget. 2016 Aug 10;7(38):61069–80. doi: 10.18632/oncotarget.11179 (PMC5308636; doi:10.18632/oncotarget.11179)
Supplement: Supplementary file 1 [file oncotarget-07-61069-s001.pdf]

Exogenous IL-33 overcomes T cell tolerance in murine acute myeloid leukemia

Supplementary Materials

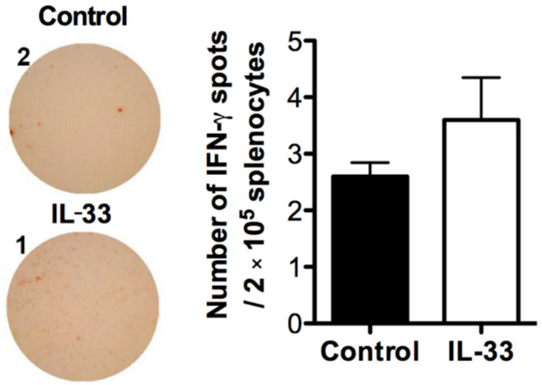

Supplementary Figure S1: IFN-γ production was barely detected without SIY peptide stimulation. Splenocytes from leukemia-bearing mouse were cultured without SIY peptide for 48 hours. Number of IFN-γ producing cells was determined by IFN-γ ELISPOT assay.

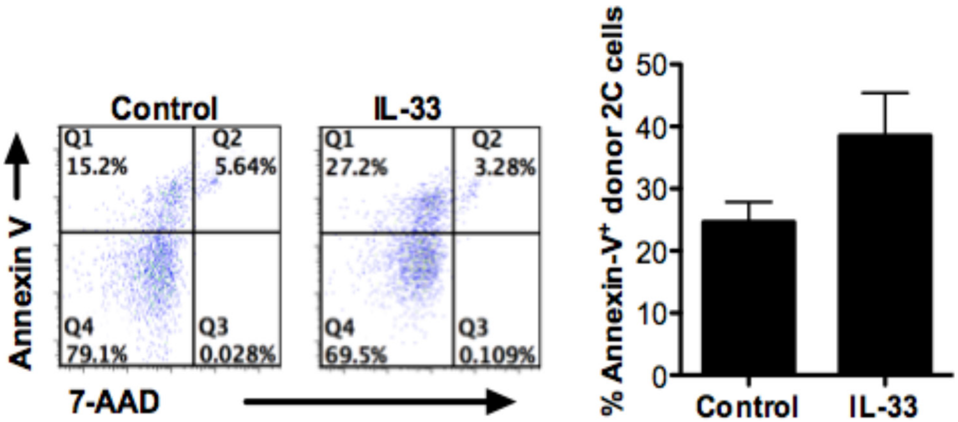

Supplementary Figure S2: The apoptotic status of adoptively transferred 2C T cells following IL-33 treatment. The apoptosis of transferred 2C T cells (1B2^{+}CD8^{+}) in spleen from C1498. SIY-bearing mice treated with IL-33 or control PBS was determined by Annexin V and 7-AAD staining. The *left* panel showed the representative flow dot plots, and the *right* panel indicated the summary of the frequency of apoptotic (Annexin V^{+}) 2C T cells.

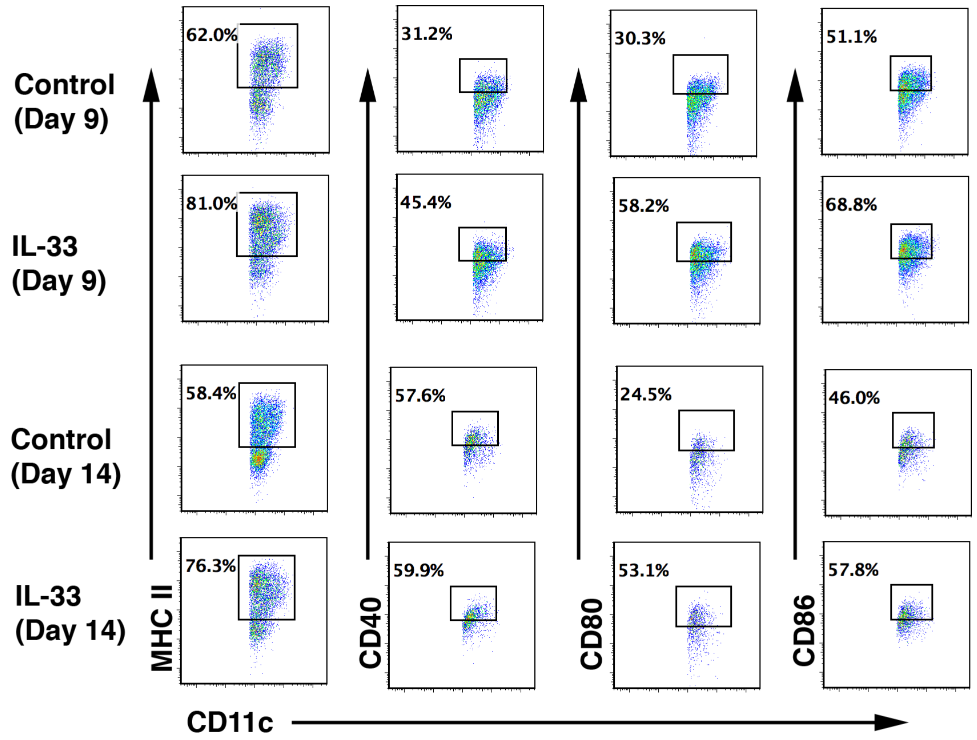

Supplementary Figure S3: Representative flow dot plots of MHC II, CD40, CD80 and CD86 positive splenic DCs in C1498. SIY bearing mice on day 9 and 14.

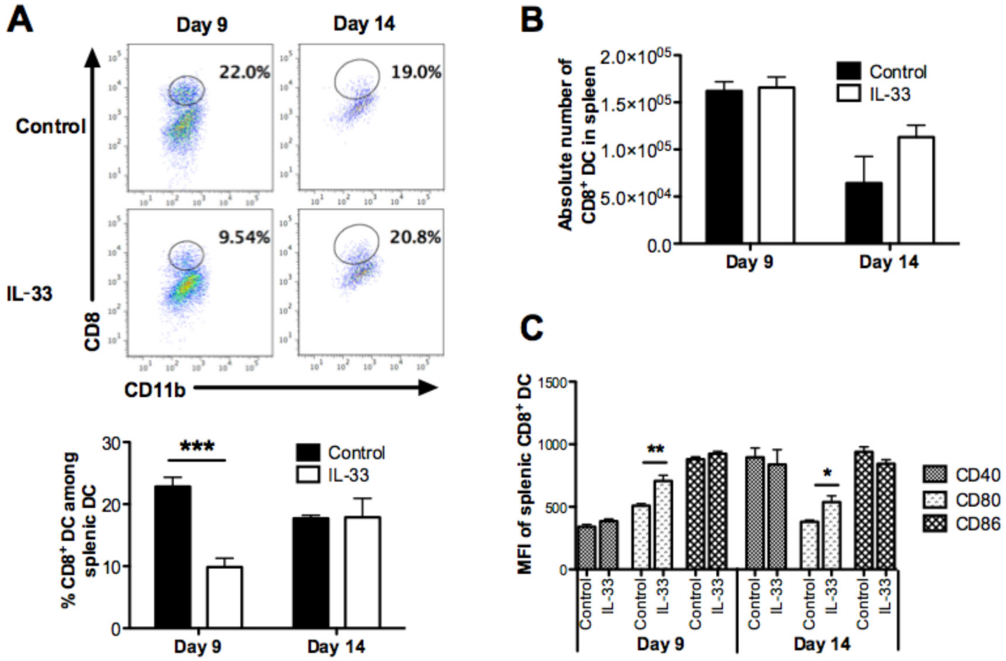

Supplementary Figure S4: The effect of exogenous IL-33 treatment on splenic CD8⁺ DCs in C1498.SIY-bearing mice. (**A**) Representative flow dot plots showed the frequency of CD8⁺ cells among splenic CD3-CD11b^{low} CD11c⁺ DCs (*upper*) that was summarized in the bar graph (*lower*, $n = 5$). (**B**) Absolute numbers of splenic CD8⁺ DCs in spleens were calculated. (**C**) The MFI of CD40, CD80 and CD86 expression on CD8⁺ DCs was determined by flow cytometry. *$p < 0.05$. **$p < 0.01$. ***$p < 0.001$.
